# SoftSearch: Integration of Multiple Sequence Features to Identify Breakpoints of Structural Variations

**DOI:** 10.1371/journal.pone.0083356

**Published:** 2013-12-16

**Authors:** Steven N. Hart, Vivekananda Sarangi, Raymond Moore, Saurabh Baheti, Jaysheel D. Bhavsar, Fergus J. Couch, Jean-Pierre A. Kocher

**Affiliations:** 1 Division of Biomedical Statistics and Informatics, Department of Health Sciences Research, Mayo Clinic, Rochester, Minnesota, United States of America; 2 Division of Experimental Pathology, Department of Laboratory Medicine and Pathology, Mayo Clinic, Rochester, Minnesota, United States of America; Indiana University, United States of America

## Abstract

**Background:**

Structural variation (SV) represents a significant, yet poorly understood contribution to an individual’s genetic makeup. Advanced next-generation sequencing technologies are widely used to discover such variations, but there is no single detection tool that is considered a community standard. In an attempt to fulfil this need, we developed an algorithm, SoftSearch, for discovering structural variant breakpoints in Illumina paired-end next-generation sequencing data. SoftSearch combines multiple strategies for detecting SV including split-read, discordant read-pair, and unmated pairs. Co-localized split-reads and discordant read pairs are used to refine the breakpoints.

**Results:**

We developed and validated SoftSearch using real and synthetic datasets. SoftSearch’s key features are 1) not requiring secondary (or exhaustive primary) alignment, 2) portability into established sequencing workflows, and 3) is applicable to any DNA-sequencing experiment (e.g. whole genome, exome, custom capture, etc.). SoftSearch identifies breakpoints from a small number of soft-clipped bases from split reads and a few discordant read-pairs which on their own would not be sufficient to make an SV call.

**Conclusions:**

We show that SoftSearch can identify more true SVs by combining multiple sequence features. SoftSearch was able to call clinically relevant SVs in the BRCA2 gene not reported by other tools while offering significantly improved overall performance.

## Introduction

Many patients at high-risk for developing cancer have a negative finding from mutation screening [1]. One possible explanation for the discordance is that the mutation is not of a single base or small insertions or deletions, but rather a large structural rearrangement (SV) that is missed when looking for smaller events. For instance, women with a family history of breast or ovarian cancer with point mutations in the BRCA1 and BRCA2 genes are clinically recognized to have a high risk of developing breast cancer. However, structural variations in BRCA1 and BRCA2 are also risk factors for the disease [2–4]. A recent study has suggested that the frequency of SV in BRCA1 and BRCA2 genes could comprise as high as 18% of all BRCA mutations [5], and many of these are likely causative of cancer susceptibility in the families in whom they were identified [6], and is recommended to be used in clinical practice [6]. Until recently, the process of SV discovery in disease genes like BRCA1 and BRCA2 required gene-specific probes to amplify and quantify the genomic DNA structure and amount, which made it difficult to identify new genes contributing to breast cancer risk though mechanisms such as SV.

Next-generation sequencing technologies offer the unprecedented capacity to characterize SVs at the genome wide scale, many of which were not possible to discover on conventional microarray platforms [7]. Three commonly used approaches to identify SVs are read depth, read-pair, and split read. Read depth approaches, such as CNVnator [8], identify changes in copy number by categorizing areas of the genome that have higher or lower coverage than expected. Read pair approaches such as BreakDancer [9] and HYDRA [10], detect SVs by utilizing mated reads that map to the reference genome with an unexpected orientation (e.g. both align to the + strand of DNA), align to different chromosomes, or that display an abnormal insert size (i.e. with a distance between mapped read-pair ends smaller or larger than expected from Illumina sequencing protocol). Finally, split-read approaches like SPLITREAD [11] and CREST [12], use single reads that initially only partially align to the reference genome. SPLITREAD requires all possible locations of reads to be mapped (a computationally expensive exercise), and then looks for areas where clusters of reads map but their mates do not (i.e. an unmated read pair referred as uRP). The unmapped read from the uRP is split into smaller fragments and then remapped to the genome to resolve the breakpoint. An alternative strategy involves the use of soft-clipped reads. Soft-clipped reads are reads where one portion of the read is mapped to the genome, but the other portion differs substantially from the reference genome at that location. CREST uses a local assembly the unmapped bases from overlapping split reads and then search the genome for their location. The advantage of this method is that it precisely defines the location of the breakpoint at base pair resolution. Its disadvantages are that it doesn’t use information from discordant read pairs to find additional support for the breakpoint and the assembled contig from the split reads must be sufficiently long enough to map uniquely to one location in the genome.

Many split-read tools do not use read-pair information and many read-pair tools do not use split-read information. SVseq2 [13] , PRISM [14], and recently DELLY[15] are exceptions. SVseq2 attempts to resolve split reads, requiring both sides of the split read to be aligned within a maximum allowable size and then looks for discordant read pairs (DRPs) to support the SV. Alternatively, PRISM looks for DRPs where only one end maps with reliable mapping, then uses these areas to attempt split-read mapping of unmapped reads at the focused loci, thus decreasing the search space for mapping fragments of reads. For these approaches, both sides of a read are required to be mapped and an additional alignment step is needed. DELLY expects multiple insert size libraries to be generated, but can operate given only one (like most sequencing experiments). It first seeks out clusters of discordant read pairs, and then looks for reads where one mate aligns but the other does not, and then uses a split-read mapping algorithm for unmapped read from the unmated pair. This assumes that the unaligned mates span the putative breakpoint, which prohibits their alignment.

To circumvent these limitations, we developed SoftSearch, which effectively uses the intrinsic information from the initial alignment process. According to the SAM/BAM format specification [16], columns 3 and 4 are the chromosome and position of the read, and columns 7 and 8 are the chromosome and position of the corresponding mate. Assuming soft clipping delineates the exact breakpoint position and direction, DRPs overlapping such soft-clipped areas should already contain the information about the type and size of SV, obviating the need for secondary alignments. Thus SoftSearch is able to detect large Insertions, large deletions, inversions, tandem duplications, novel sequence insertion locations, and chromosomal translocations.

## Methods

### Breakpoint Detection

**Figure 1:**
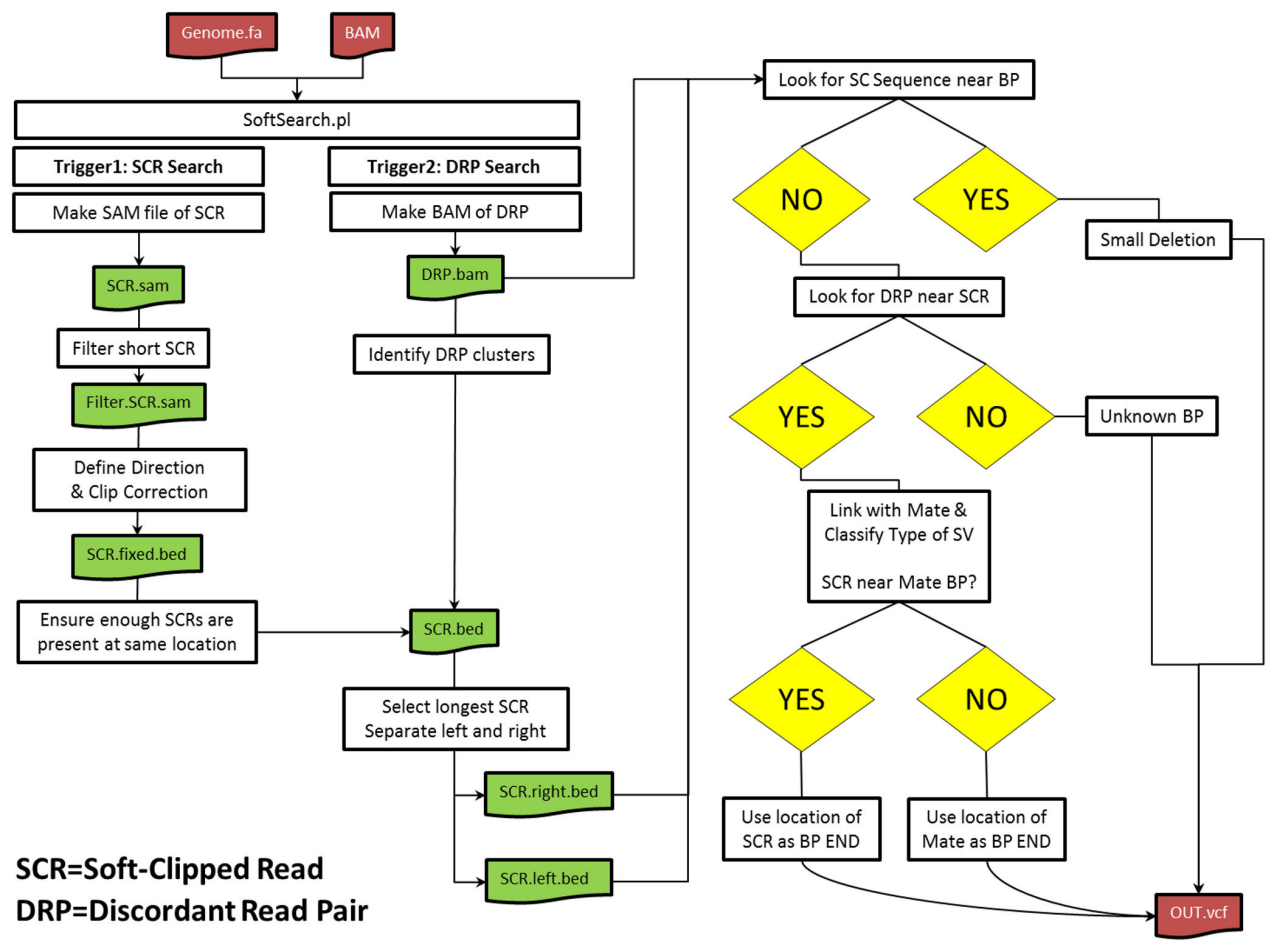
The general strategy for SoftSearch. A) Left clipped reads are defined as where the clipped portion of the read is at a smaller genome coordinate than the opposite end (opposite for right clipping). For a left clipped read located on the “+” strand, SoftSearch looks upstream for a discordant read pair where the read is oriented in the “-” direction. The orientation and location of the mate is where SoftSearch links the first region to. To increase the likelihood of exactly detecting the breakpoint, it then looks upstream for a right clipped read cluster. If none is found, then the default breakpoint location is the discordant read mate location; otherwise it is the position of soft clipping at the right clipped read. B) SoftSearch determines discordant read pairs by their insert size and orientation and places them in a temporary BAM file. It also reads the input BAM file for soft clipped reads and converts them to a BED file. Overlapping soft clip locations are counted to identify putative breakpoints, and then queried against the discordant read pair bam file for properly oriented supporting reads, which are then output in VCF format.

The general strategy for SoftSearch is given in Figure **1**. SoftSearch requires only two inputs: a BAM file of aligned reads and the genome to which the reads were aligned. BAM files can come from any aligner capable of encoding soft-clipping into reads (e.g. BWA, Novoalign, Bowtie, Mosaik, etc.). If not supplied with any arguments, or the –h flag, SoftSearch will display the available parameters, a brief explanation of those parameters, and usage statement. The distribution of insert sizes is computed from a subset of properly paired reads. The user can specify the number of standard deviations away from the mean to define insert sizes that are larger or smaller than expected. After removing unmapped reads and PCR duplicates, a new SAM file is created for soft-clipped reads excluding any reads that contain the “#” value in the quality string – which indicates poor quality sequencing. This filtering step is essential to exclude thousands of false positives resulting from poor quality reads (data not shown). At each genomic location, the number of reads with either left- or right- soft clipped bases is computed. Left-clipped bases are defined when a read on the minus strand has soft clipping at the 3’ end of the read, or alternatively the 5’ portion of the read if on the positive strand (Figure **S1**). Soft-clipped reads with more than 5 unmapped bases are passed through for further analysis. 

Next, soft-clipped reads supporting a break point event are combined if the left/right orientation is in the same direction. A putative breakpoint is defined when there is at least x soft-clipped reads beginning at position y. The number of soft-clipped reads that must be co-located is a user defined parameter – requiring fewer events increases the detection of false positives, but increases the sensitivity. Because small deletions can also cause an accumulation of soft-clipped reads, we search the reference genome sequence in the region where the soft-clipped bases are located. Using the longest soft-clipped read as the query sequence, we calculate how many edits are needed to transform the query sequence into a match in the reference sequence (a.k.a. the Levenstein distance; LevD) around the location in which it was observed. If this “local LevD” is less than 5% we consider the soft-clipped bases as matched to the reference genome then the event is a small deletion in which case is excluded from the remaining analysis. Otherwise, the event is considered a putative structural variant and sent for further processing.

### Structural Variant Calling

Each putative breakpoint is searched for a minimum number DRPs at a given distance that support a SV. For a left-clipped breakpoint, SoftSearch looks in the DRP BAM file for a read that aligns upstream on the “-” strand. This strand information is crucial to ensure that the DRP crosses the junction formed by the split-read. Using the mate location as a starting point, SoftSearch looks for soft-clipped reads near the discordant read-mate (the other discordant read in the pair). If the mate is oriented on the “+” strand, then SoftSearch will look upstream the same number of bases to identify a right-clipped read. If no additional split-read is found, then the position of the mate is used as the putative breakpoint. SoftSearch then calculates the “distal LevD” to determine whether or not the sequence that is soft-clipped is actually present near the proposed mate breakpoint site. This differs from the local LevD which looks for the soft-clipped bases near where they were initially observed. The LevD in this instance is more of an annotation, which the user can decide later whether or not to trust SVs with low LevD scores. The orientation and insert size of the DRP is used to annotate the type of SV present, either as insertion (insert greater than 0, but less than expected), deletion (insert larger than expected), inversion (reads on same strand), tandem duplication (read1 begins before read 2), or inter-chromosomal translocation (Figure **S1**). As an aside, SoftSearch is also capable of using only discordant read pair information, rather than leveraging the soft-clipping information. In this case, the average location of the discordant read pair clusters are treated as soft clipped reads and the breakpoint is flagged. This type of analysis would be well suited for Mate-pair analysis, since the biotin junction (a part of the library preparation) can make sequences difficult to resolve accurately, causing large number of soft-clipped reads. Variants are exported in VCF4.1 format, with several annotations that can be used for post-process filtering. To do all of this extracting and merging effectively, SoftSearch heavily leverages the BEDTools [17] and samtools [16] packages. SoftSearch is implemented in perl with BASH system calls to BEDTools and samtools. SoftSearch can be downloaded from http://bioinformaticstools.mayo.edu/.

### Simulation Data set

To estimate the sensitivity and specificity of SoftSearch and other tools, we created an artificial genome using Genome_Smasher (v.3871; http://code.google.com/p/genome-smasher/). Structural variants inserted into the synthetic genome were based on gold-standard calls for the HapMap sample NA12878 [18]. These variations included 616 deletions, 265 duplications, and 84 insertions. Random sequences were used to fill in the insertions since the actual sequences were not available. Since the number of modifications was limited, we also supplemented random variations until the sample contained the following SVs: 1227 deletions, 526 tandem duplications, 424 insertions, 1750 inversions, and one inter-chromosomal translocation. Inversions ranged between 1kb to 50kb, Insertions were between 1kb-10kb, and a chromosomal translocation between chr19 and 21. More simulation datasets at 4x and 40x coverage are presented on the SoftSearch website (https://code.google.com/p/softsearch/wiki/Performance). 

### Real Data sets

To further test the performance of the variant detection methods on non-simulated data, we ran experiments from 3 different experimental designs: a 40X whole genome, a 4X whole genome, and a 2000X custom capture panel. All datasets are annotated with a set of experimentally validated structural variants. The 40X whole genome sequence data for the HapMap sample NA12878 was downloaded from ftp://ftp-trace.ncbi.nih.gov/1000genomes. The 4X genome, (also a HapMap sample) was downloaded from (ftp.1000genomes.ebi.ac.uk/vol1/ftp/data/NA18507/alignment/) as a BAM file that had already been aligned using BWA (0.5.9-r16) to an alternative version of the human genome (ftp.1000genomes.ebi.ac.uk/vol1/ftp/technical/reference/phase2_reference_assembly_sequence/hs37d5.fa.gz). The experimentally validated positions were downloaded from the 2013-07-23 release of the Database of Genomic Variation (http://dgv.tcag.ca/dgv/app/home). Finally we used an in-house targeted custom capture of 122 DNA repair genes sequenced at about 2000X coverage (manuscript in preparation). The 40X HapMap sample was aligned to a previous assembly of the human genome (hg18), but the other two datasets were aligned to the hg19 assembly. To be consistent with the assembly, we converted the NA12878 sample back to FASTQ to using PICARD (http://picard.sourceforge.net) and aligned to hg19 using Novoalign (v2.08.01) with the following non-default command line parameters: -x 5 -i PE 425,80 -r Random --hdrhd off -v 120 -c 12. The “--hdrhd” parameter disables the checking of identity between headers in paired end reads, “-x” is the gap extension penalty, “-PE 425,80” is the expected insert size distribution, “-r Random” randomly allocated multi-mapped reads equally between identical positions, “-v 120” sets the structural variation penalty, and “-c 12” is the maximum number of threads to use. 

### Methods Comparison and Variant Calling Accuracy

In this manuscript we compare structural variant results from five methods: SoftSearch, BreakDancer version 1.1_2011_02_21 [19], CREST [12], DELLY [15], and SVseq2.2 [13]. No version numbers are available for CREST or DELLY. For most tools, there were few parameters that could be changed modulate the sensitivity, in which case only the default parameters were used. The exceptions were SoftSearch and BreakDancer. We ran the analysis using two parameter settings for BreakDancer and three settings for SoftSearch to show how these settings influence the results. For clarity of the results, we have indicated the parameter settings in the results of each analysis. There were two settings used for BreakDancer: BreakDancer_1 refers to the default settings, whereas in BreakDancer_2 we increased the insert size to call a discordant read pair from 3 to 4 standard deviations and increased the minimum number of reads supporting an event from 2 to 5. SoftSearch modifications were as follows: In SoftSearch_1 we decreased the minimum number of softclipped reads from 5 to 2, decreased the number of spanning reads from 5 to 0, and decreased the insert size to call a discordant read pair from 6 to 4 standard deviations. SoftSearch_2 is default parameters. We also included SoftSearch_3 which was designed to mimic the BreakDancer default parameters where we did not require softclipped read support and looked for events with only two supporting read pairs with an insert size greater than 3 standard deviations. All metrics for time and memory estimates are the average of two repeated runs.

To assess the performance of each application, we use the closestBed tool in the BEDTools package[17] to find out how close predicted breakpoints were to the nearest true (i.e. experimentally characterized or spike-in) breakpoints. If a predicted breakpoint was within the normal insert size range (less than 600bp) to the true breakpoint, the prediction was considered a true positive event. False positives were any predicted breakpoint not within the acceptable range, and false negatives are any breakpoint in the real or simulated dataset that were not identified by the breakpoint detection tool. We define precision as TP/(TP+FP) and recall as TP/(TP+FN), where TP=true positive, FP=false positive, and FN=false negative. To summarize the results from the simulation data, we use the F-score: 2*(precision*recall)/(precision+recall).

## Results

### Simulated Whole Genome

Precision and recall cannot be measured from the real dataset since the entire complement of true positive variations is not known. Therefore, to test the precision and recall of various algorithms in a realistic scenario, we generated a simulated genome based on real structural variants identified in the HapMap Sample NA12878. Mills et al. [18], have previously reported a “gold standard” set of validated duplications, deletions and inversions in this sample. However, the numbers of validated breakpoints were limited, so we also supplemented known variations with random variations. The final number of breakpoints used in the synthetic genome was 1227 deletions, 526 tandem duplications, 424 insertions, 1750 inversions, and one inter-chromosomal translocation. A single translocation was simulated, since these types of variations are generally regarded as the easiest to detect. 

SoftSearch was able to recall the largest number of true positive SV calls (i.e. those called by the variant detection algorithm that are truly present in the synthetic genome). The results are shown in Table **1**. SoftSearch was able to identify 89-91% of the simulated structural variant breakpoints (regardless of parameter setting), whereas SVSeq2, DELLY, BreakDancer, and CREST were comparable at recalling 80%, 76%, 73-74%, and 68%. CREST and BreakDancer_2 had the highest precision at 99% and 100%, followed by SVSeq2, DELLY, BreakDancer, and SoftSearch (all between 84-97%). In terms of computational speed, SVSeq2, SoftSearch and BreakDancer were by far the fastest on the 40X simulated genome dataset; completing in fewer than 5.4, 8, and 13 hours, respectively. DELLY completed the analysis in less than 72 hours, but CREST required 20-30 times longer than the two fastest algorithms. Note that the simulated data might have some intrinsic characteristics that might be more favourable to some applications than others. We observed a somewhat different ranking when analysis real datasets. SoftSearch and BreakDancer used less than 0.6GB, whereas other tools used 3.2-6.2GB (Table **1**).

**Table 1:** Summary Statistics from the Synthetic Whole Genome (40X).

	**Break Dancer_1**	**Break Dancer_2**	**Soft Search_1**	**Soft Search_2**	**Soft Search_3**	**Crest**	**Delly**	**SVSeq2**
**TP**	2,907	2,884	**3,558**	3,483	3,527	2,686	2,972	3,154
**FN**	1,022	1,045	371	446	402	1,243	957	775
**FP**	385	3	666	58	601	21	381	176
**Precision**	0.88	**1.00**	0.84	0.98	0.85	0.99	0.89	0.95
**Recall**	0.74	0.73	**0.91**	0.89	0.90	0.68	0.76	0.80
**F-score**	0.81	0.85	0.87	**0.93**	0.88	0.81	0.82	0.87
**CPU (h)**	7.56	--	--	13.06	--	162.64	72.16	**5.37**
**Memory (GB)**	**0.19**	--	--	0.58	--	5.40	6.21	3.18

TP=True positive; FN=False negative; FP=False positive; Bold indicates best result.

### SV Detection in Whole Genome Sequencing

**Figure 2:**
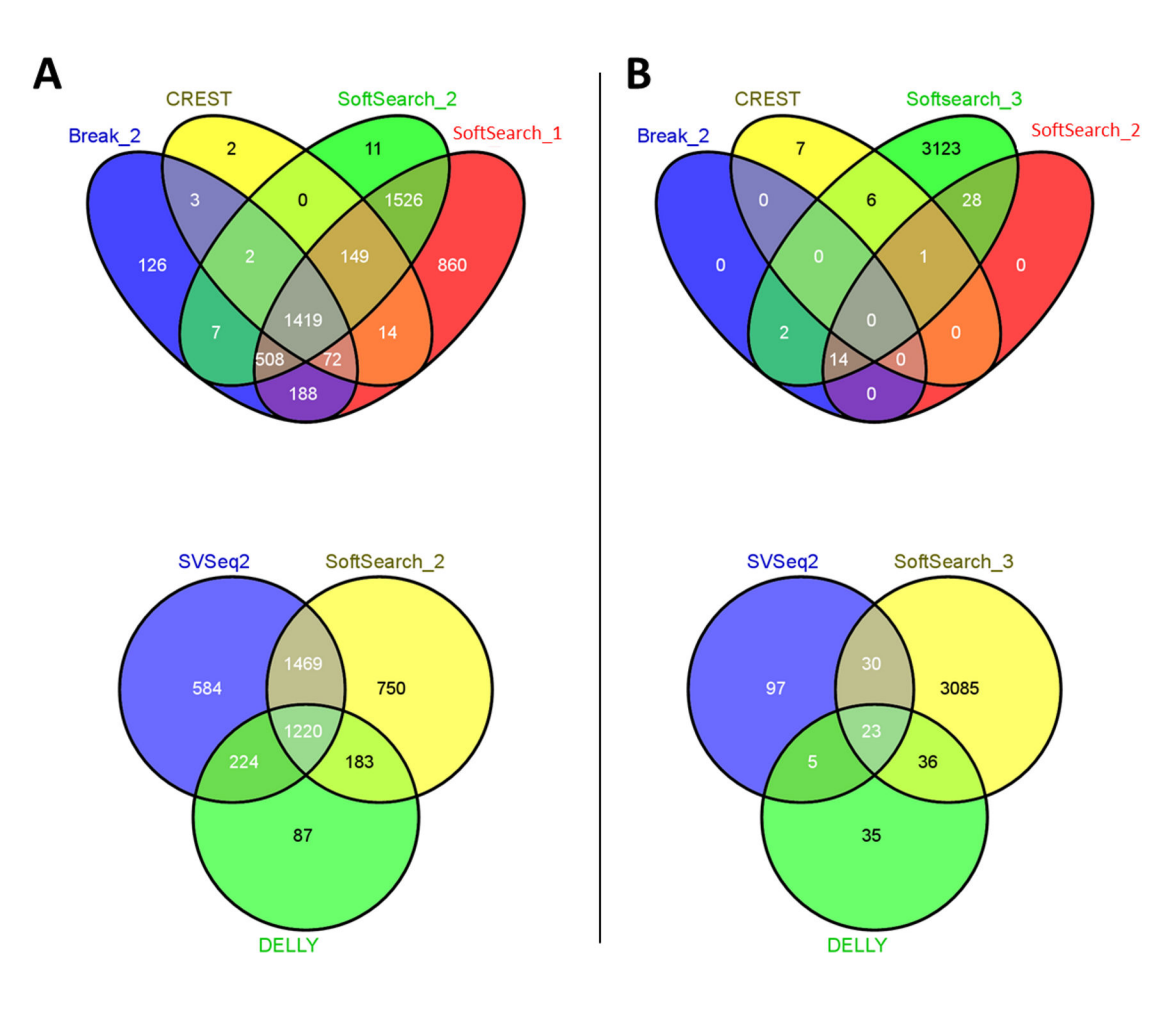
Overlap of true positive calls for the NA12878 and NA18507 datasets.

We attempted to recall the breakpoints of known structural variants that have previously been validated in the HapMap NA12878 sample [18]. This sample was sequenced on an Illumina HiSeq (2.4 billion 100 bp paired end reads). SoftSearch recalled the most true positive results (n=3,622-4,736), followed by SVSeq2 (n=3,497), and BreakDancer (n=2,325; both settings) (Figure **2A**). As one would expect, many of the SVs (74%) were identified by more than one tool and 19% by all tools. 

 SoftSearch, SVSeq2, and BreakDancer were the most similar, respectively identifying 72-94%, 70%, and 46% of the discoverable breakpoints independently. There were no differences in results between BreakDancer_1, and BreakDancer_2. Note that the false positive detection rate could not be determined in this sample since, true but un-validated structural variants might be present in these HapMap samples. Instead, we assume that the number of false positives would be somewhat similar to that of the simulated data set. CREST and SVSeq2 took the longest amount of CPU time at 255 h and 425 h, respectively. SoftSearch completed the analysis in less than 90 hours using only 0.6 GB memory. BreakDancer was faster at 9 hours and 0.7GB memory. 

We used a similar approach to evaluate the performance on a low coverage HapMap NA18057 sample as well. SoftSearch identified the most true positive breakpoints, followed by BreakDancer, then DELLY and CREST (Figure **2B**). Strikingly, SoftSearch_3 found 20-fold more true breakpoints than SVSeq2 (n=3,174 versus n=155). Only 43 SV were identified using SoftSearch’s default parameters, which is not surprising since it requires 5 reads to be softclipped even though most of the genome is only sequenced at 4X or lower. There were no differences in results between BreakDancer_1, and BreakDancer_2. In this analysis, SoftSearch took the longest time to complete (60 CPU hours) compared to less than 5.3 hours for all other tools, which is expected given the relatively large number of breakpoints identified. SoftSearch, CREST, and BreakDancer all used < 1.4GB of memory whereas DELLY used 6GB. 

### Structural Variations in the BRCA2 Gene Identified in Custom Captures

**Figure 3:**
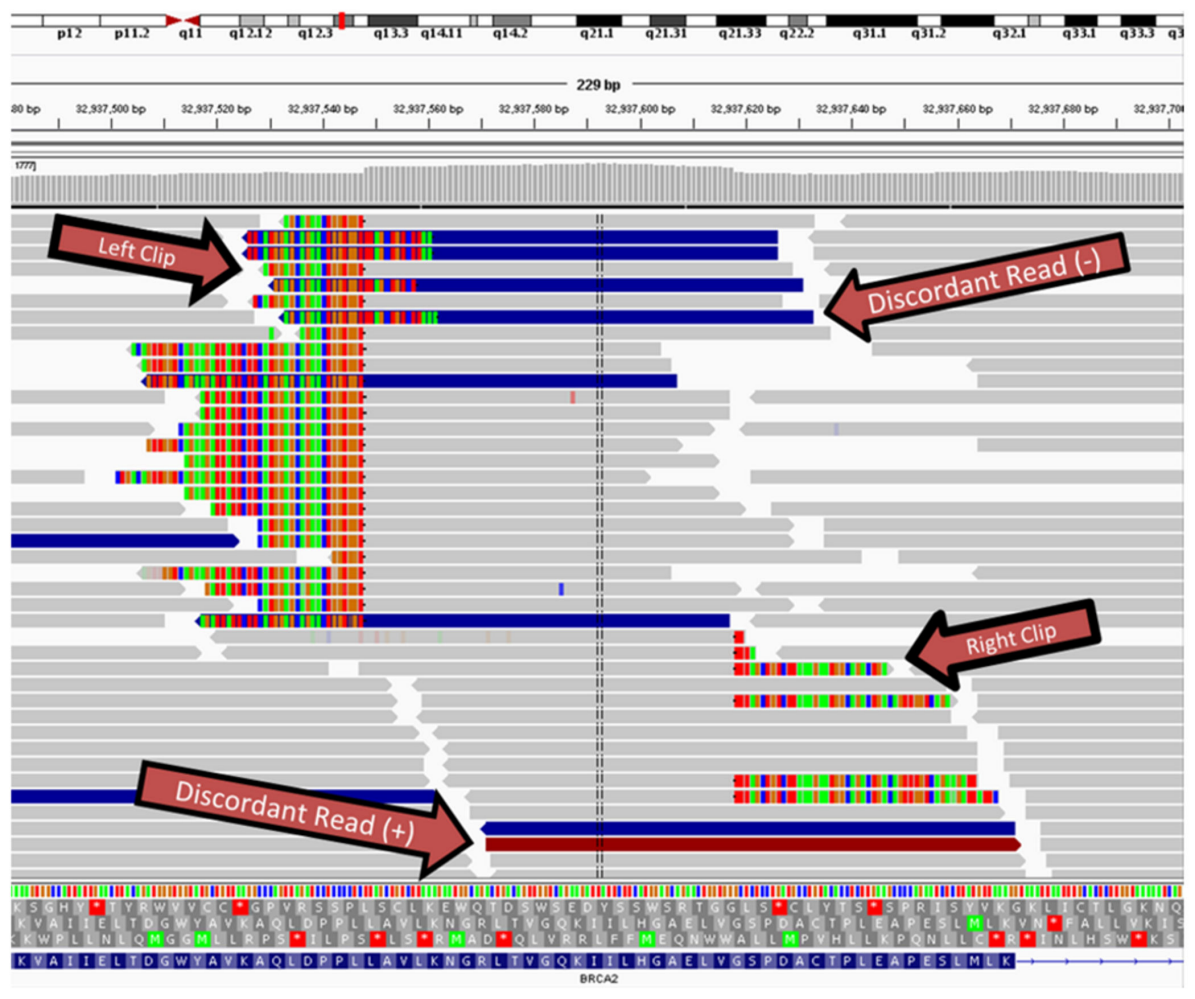
Example IGV screenshot of a 71bp tandem duplication in the BRCA2 gene identified by SoftSearch. Discordant reads are blue (plus strand) or red (minus strand). Soft clipped bases appear as multicolour “rainbows”.

We also used high coverage custom capture experiments to identify how SV detection performs on high coverage datasets (~2,000X of 122 genes). CREST, SVSeq2 and SoftSearch identified a 6.6kb heterozygous deletion of the BRCA1 gene, but CREST and SVSeq2 missed a 71bp tandem duplication in the BRCA2 gene (Figure **3**), both of which were experimentally validated by Myriad Genetics (www.myriad.com). SVSeq2 also made several false positive calls for each sample (data not shown). BreakDancer and DELLY did not detect any SVs in the target region.

## Discussion

SoftSearch is a new breakpoint detection tool for paired-end next generation sequencing instruments that uses multiple sequence features to infer breaks point to characterize location and type of structural variants. SoftSearch uses less memory, has a fast and consistent run time, and is more sensitive than other tools. SoftSearch does not require additional alignment steps so it can easily plugged into existing workflows. The results obtained from the analysis of the whole genome simulations datasets, showed that SoftSearch is able to identify more accurate breakpoints than other tested tools. The higher sensitivity of SoftSearch was also observed in high and low coverage HapMap samples (40X and 4X coverage), and a high coverage 122 gene custom capture dataset. The significantly higher recall obtained with SoftSearch on the 4x coverage HapMap highlights that the SoftSearch strategy of combining multiple sequence features to call breakpoint is more effective than relying only on a single feature. Compared to DELLY that operates on similar principle, SoftSearch’s count requirement for these features is lower. DELLY must first identify a significant cluster of paired end reads and then the breakpoints of those clusters are refined using the split reads. SoftSearch works in the opposite direction, first identifying anywhere in the genome softclipped reads occur at exactly the same point. If there are only a few read pairs supporting the event, then the discordant reads cannot be distinguished from background and the variant is missed. In the case of SoftSearch, anywhere at least two discordant read pairs support a split read will be reported. Finally, SVSeq2 was the most comparable to SoftSearch in terms of recall. One limitation of SVSeq2 is that it will only annotate variants as insertion or deletion, even when breakpoints overlap with known inversions (Table **S1**). 

The analysis of a high coverage (at 2,000X) 122 genes capture experiment, although done on a very limited scale (12 samples), shows that SoftSearch was able to detect a 71bp tandem duplication in the BRCA2 that was not called by either BreakDancer or CREST. This result again highlights SoftSearch’s sensitivity but also emphasizes its ability to process custom capture experiments. This is of particular importance since many more exomes or custom capture experiments than genomes have been sequenced, many of which have not yet been exploited for SV discovery.

As a final point of this discussion, we would like to stress that each of the tested algorithms is capable of identifying correctly breakpoints and structural variants missed by the other tools. This suggests that a combined approach that could summarize the results of multiple structural variant callers would result into the highest possible sensitivity. 

## Supporting Information

Figure S1 **Strategy for detecting SV from right and left clipped reads.**
(TIFF)

Table S1 **Breakdown of SV call from each tool for each sample.**
(XLSX)
