# Correction: SoftSearch: Integration of Multiple Sequence Features to Identify Breakpoints of Structural Variations

**DOI:** 10.1371/journal.pone.0093027

**Published:** 2014-03-14

**Authors:** The PLOS ONE Staff

The following information is missing from the Funding section: The funding source for this project is internal to Mayo Clinic and was provided by the Center for Individualized Medicine at Mayo Clinic. No additional external funding was received for this study. The funders had no role in study design, data collection and analysis, decision to publish, or preparation of the manuscript.
