# Supplementary figures and images for: SoftSearch: Integration of Multiple Sequence Features to Identify Breakpoints of Structural Variations
Source: PLoS One. 2013 Dec 16;8(12):e83356. doi: 10.1371/journal.pone.0083356 (PMC3865185; doi:10.1371/journal.pone.0083356)

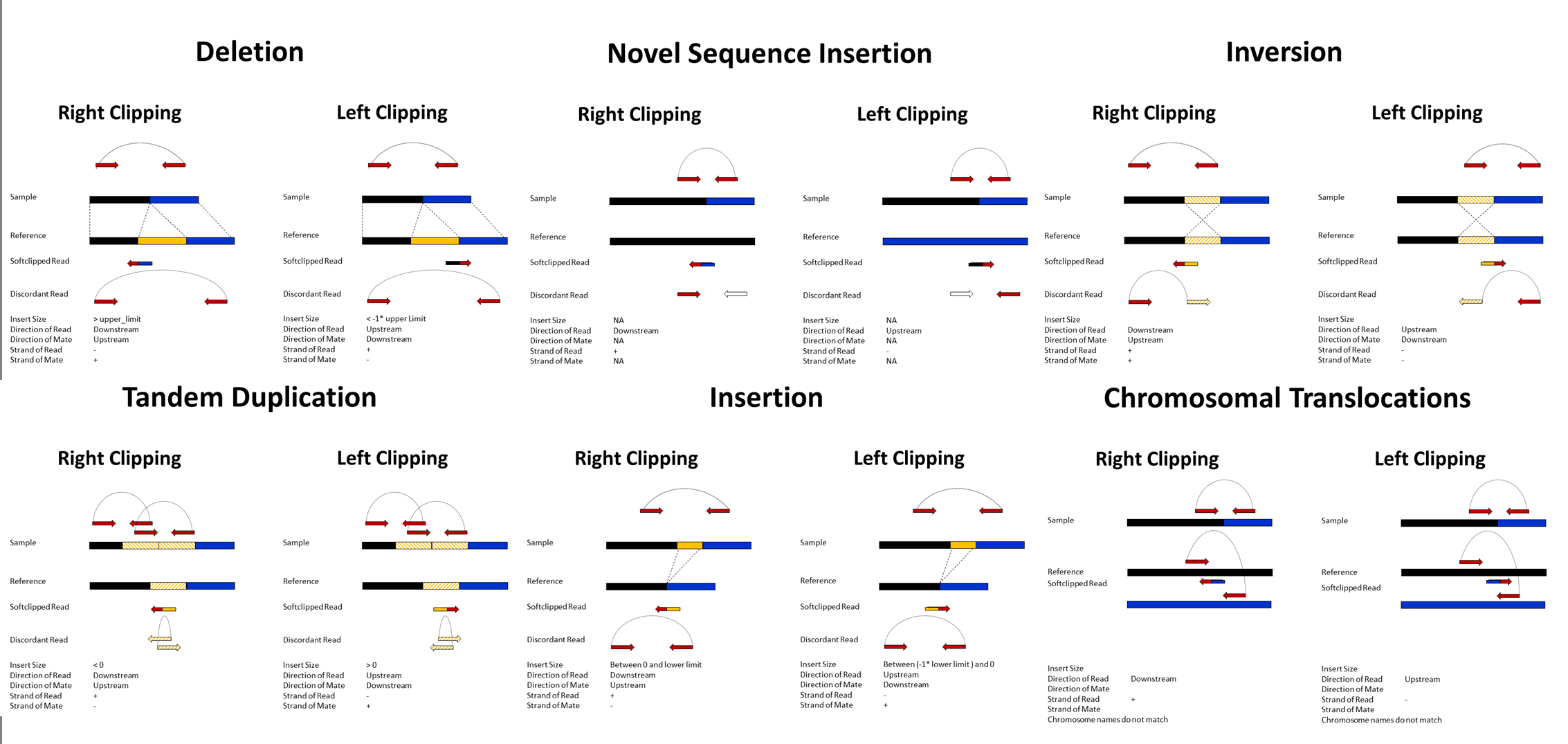

Supplement: Figure S1 — Strategy for detecting SV from right and left clipped reads. (TIFF) [file pone.0083356.s001.tiff]
